# Coronal native limb alignment: establishing reporting standards and aligning measurements of key angles

**DOI:** 10.1530/EOR-2024-0119

**Published:** 2025-08-04

**Authors:** The Scientific Committee from the Personalized Arthroplasty Society (PAS)

**Affiliations:** ^1^ Personalized Arthroplasty Society, Atlanta, Georgia, USA

**Keywords:** knee, arthroplasty, personalized, coronal, limb, alignment

## Abstract

- The main goal of a successful total knee arthroplasty is to relieve pain and restore function. While mechanical alignment provides excellent long-term implant survivorship, clinical and functional outcomes remain less than ideal.
- As a result, the focus has gradually shifted to a more personalized surgical approach based on the patient’s specific characteristics.
- There is a pressing need for agreement on definitions of key terms to standardize limb alignment measurements and improve understanding and communication within the field.
- This work aims to clarify the concept of native limb alignment, outline how it is measured, and propose a standardized terminology to describe it.

## Introduction

The main goal of a successful total knee arthroplasty (TKA) is to relieve pain and restore knee function by providing the patient with a stable and well-aligned joint. Correct restoration of limb alignment significantly impacts long-term implant survivorship and clinical outcomes.

For decades, a stable knee has been considered a neutrally aligned knee, achieved by positioning both the femoral and tibial components perpendicular to the mechanical axis (1). While this approach has demonstrated excellent long-term implant survivorship, clinical outcomes often remain suboptimal (2, 3, 4). Recently, an increasing number of surgeons have questioned whether alternative surgical alignment strategies might address the persistent high rates of patient dissatisfaction following TKA, which affects almost one in five patients (2, 5).

Two primary alignment concepts – limb alignment and component alignment – often overlap and are used interchangeably. Studies in the last decade have revealed substantial complexity and variability in native limb alignment. These findings suggest that a one-size-fits-all approach may only be optimal for a minority of patients (6, 7, 8, 9). Consequently, the focus has shifted from ensuring implant survivorship alone to improving clinical and functional outcomes through personalized approaches tailored to patient-specific anatomical characteristics.

The high variability in native limb alignment, combined with the complex native anatomy of the femur, patella, and tibia, as well as different knee phenotypes and assessment methods, has sparked significant debate within the orthopedic community. Alignment remains a relative concept, as it depends on the frame of reference, anatomical landmarks, and axes being used for assessment. Further complicating matters, pre-surgical alignment and presumed native alignment can be difficult to compare with post-surgical results, as alignment may vary significantly even within the same patient (10, 11).

Efforts to standardize definitions and terminology face challenges due to the ambiguities regarding which lines, axes, or planes are being used, what anatomical angles are referenced, which measurements are necessary to describe limb alignment, and which imaging modalities are used (7, 9, 12, 13).

In response to these challenges, the Personalized Arthroplasty Society (PAS) formed a Scientific Committee workgroup comprising international experts to define key concepts related to limb alignment. This initiative aims to clarify the current deficiencies by establishing clear definitions and terminology to reduce confusion in the field. The workgroup began with an initial brainstorming session followed by monthly meetings where various alignment concepts were thoroughly discussed. A literature review was conducted to compile existing knowledge and identify gaps in understanding. Initial definitions were drafted, rigorously scrutinized, and refined by the experts, followed by review from an international panel of PAS specialists.

The overarching goal of this initiative is to clarify the concept of native limb alignment, outline its measurement, and propose a standardized terminology to improve communication, research, and clinical practice.

## Definitions of native limb alignment and the angular geometry of the knee

Limb alignment patterns, as observed in the coronal plane during standing, are typically described based on the relationship between the knee and the load-bearing axis. (Please note that a line is defined by two points in a plane, and an axis is usually a line around which an object rotates. For simplicity, the load-bearing axis will be used to describe alignment.) This axis extends from the center of the hip joint to the center of the ankle joint. Historically, the term mechanical axis of the lower limb was often used interchangeably with the load-bearing axis due to the predominance of mechanical alignment techniques.

However, the mechanical axis of the lower limb is a rigid subset of the load-bearing axis, requiring specific anatomical conditions to be met. For a knee to align precisely with the load-bearing axis and to be in neutral alignment, two prerequisites must be satisfied:

- The distal femoral and proximal tibial joint lines must be perfectly parallel.
- The load-bearing axis must fall precisely through the center of the knee joint.

In this scenario, the distal femoral anatomy perfectly compensates for the proximal tibial anatomy without additional coronal plane laxity. Recent studies have highlighted deviation from this rigid construct, emphasizing concepts such as constitutional varus and patient-specific phenotypes. These findings highlight the variability in native knee alignment, which cannot be fully captured by the mechanical axis of the lower limb. Therefore, this work advocates for using the term *load-bearing axis* instead of mechanical axis of the lower limb for two reasons:

- The load-bearing axis encompasses the full spectrum of alignment variability, tolerating deviations on both sides of the coronal plane.
- It provides a more inclusive framework to describe native alignment patterns (14).

**Figure 1:**
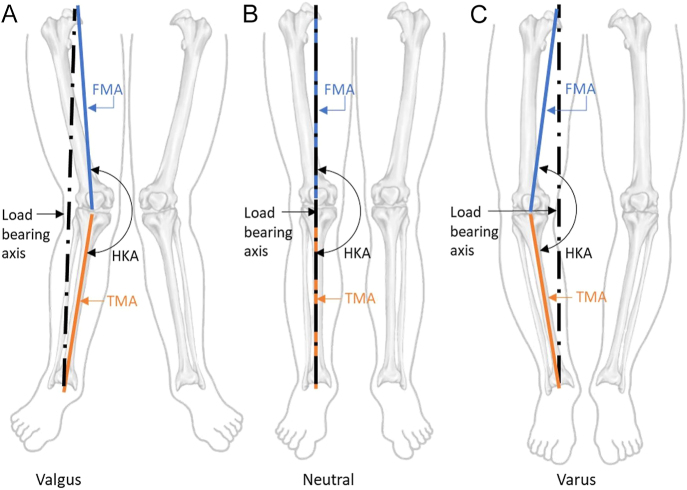
Valgus alignment: knee center is medial to the load-bearing axis (HKA >180°). Neutral alignment: knee center is on the load-bearing axis (HKA = 180°, 0° of deformity). Varus alignment: knee center is lateral to the load-bearing axis (HKA<180°). Mechanical HKA (mHKA) measures the angle between the FMA (connecting the hip center to the intercondylar notch center) and the TMA (connecting the tibial plateau center to the ankle joint center).

A knee centered on this load-bearing axis is considered neutrally aligned, as illustrated in Fig. 1. If the knee deviates laterally, forming an outward angle between the femur and tibia, it is classified as varus. Conversely, if the knee deviates medially, forming an inward angle, the alignment is defined as valgus.

## Measurement methods and limitations

Standard approaches to measuring limb alignment rely on standing full-length frontal radiographs of the lower limb, which remain the clinical gold standard for clinical evaluation. These images depict the projections of the femoral and tibial bones in the coronal plane and have significantly influenced our understanding of alignment patterns.

However, limitations exist when interpreting two-dimensional (2D) radiographs. CT-based analyses have revealed that the true distal femoral reference points correlate with rotational parameters such as the posterior condylar line, the anatomical and surgical trans-epicondylar axes, and Whiteside’s line (15, 16). Consequently, traditional 2D radiographic methods cannot fully appreciate the rotational dimension of the distal femur, reducing it to a single-line projection.

### Paley and Cooke methods

The most widely referenced methods for describing alignment in the frontal plane are those published by Paley (17) and Cooke (18). While both methods analyze the same underlying anatomical features, they differ in terminology. Each approach utilizes similar axes, lines, and tangents to describe the femur, tibia, and joint surfaces:

Currently, alignment between the femur and the tibia at the knee is most commonly represented by the hip-knee-ankle (HKA) angle, which reflects the sum of the angular contributions of the distal femur, joint surfaces, and proximal tibia. Specifically:

- For the distal femur: mechanical lateral distal femoral angle (mLDFA) or condylar-hip angle (CH).
- For the joint surfaces: joint line convergence angle (JLCA) or condylar-plateau angle (CP).
- For the proximal tibia: mechanical medial proximal tibial angle (mMPTA) or plateau-ankle angle (PA).

Historically, Cooke & Sled (18) formalized the mathematical relationship as:

HKA = CH + CP + PA

While this nomenclature may appear complex, the Paley terminology has gained widespread acceptance, particularly in clinical and research settings. For consistency and clarity, therefore, the concept of angle complementarity will serve as the foundational principle for unifying these terms.

## Standard radiographs used for assessing limb alignment

Standardized radiographic imaging is critical when evaluating limb alignment, as radiographs provide two-dimensional representations of a three-dimensional anatomical structure. Structural variations, including femoral bowing, femoral neck anteversion, and torsional anomalies, can affect measured axis values and introduce inherent measurement errors (19). While these errors are inherent to radiographic interpretation, they can be minimized through consistent image acquisition techniques. Standardization is indispensable for accurate operative planning, reproducible data interpretation, and constructive professional dialog. Importantly, minimizing variability in image acquisition is as crucial as the radiographic views themselves. To comprehensively evaluate knee alignment, three standard radiograph views are required: the anteroposterior (AP) view, lateral view, and sunrise (skyline) view.

### Anteroposterior (AP) view

**Figure 2:**
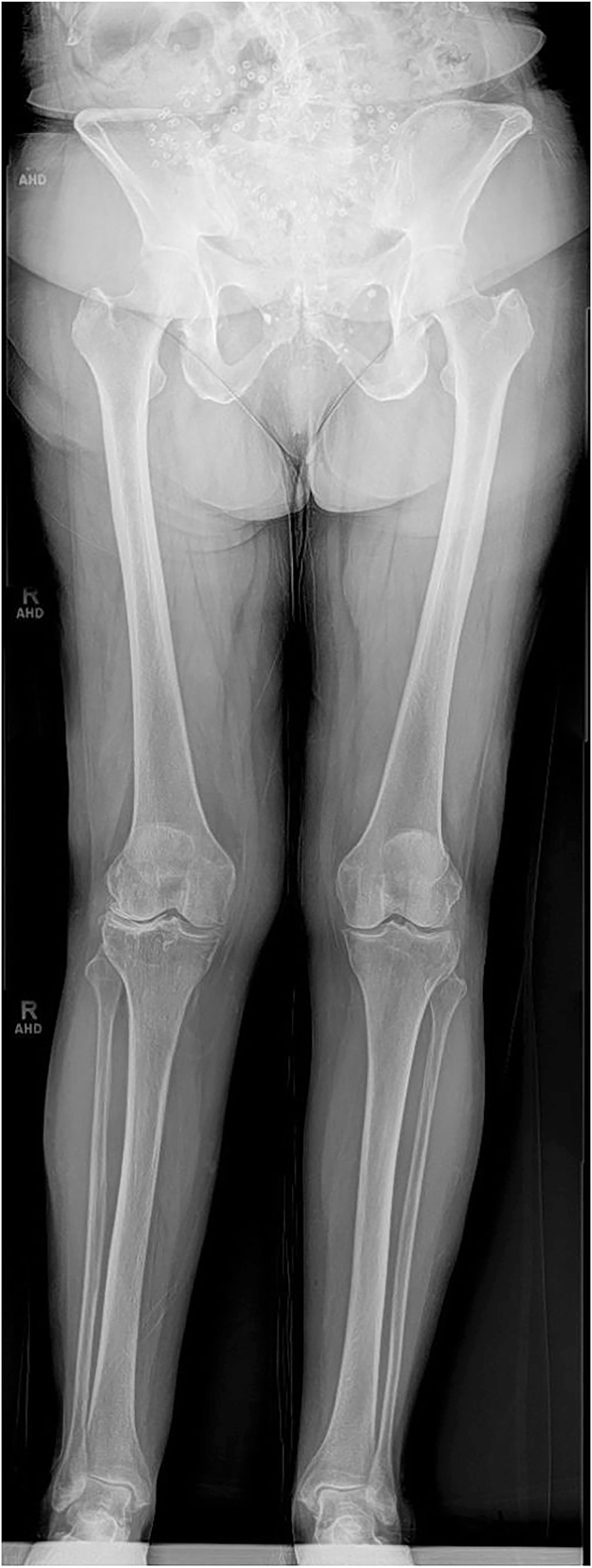
Coronal plane imaging. Full-length radiograph while the patient is bearing weight on the limb.

To accurately evaluate coronal plane alignment, full-length, weight-bearing radiographs are essential, as illustrated in Fig. 2 (20, 21, 22, 23). These images are critical for assessing the degree of alignment deviation under body weight and identifying arthritic changes. Patients should maintain a consistent standing, weight-bearing position with equal distribution of body weight on both limbs.

The radiographic beam should be centered on the knee joint line. When obtaining digitally stitched long-leg images, the beam must be directed sequentially at the joint line, and the proximal and distal section of the limb. Consistency in patient position and weight distribution throughout the imaging process is crucial to minimize measurement errors.

Although the Rosenberg view (weight-bearing radiographs with 45° of knee flexion) is valuable for assessing arthritic changes, it is not typically required for determining limb alignment and is therefore considered supplementary.

### Lateral view

**Figure 3:**
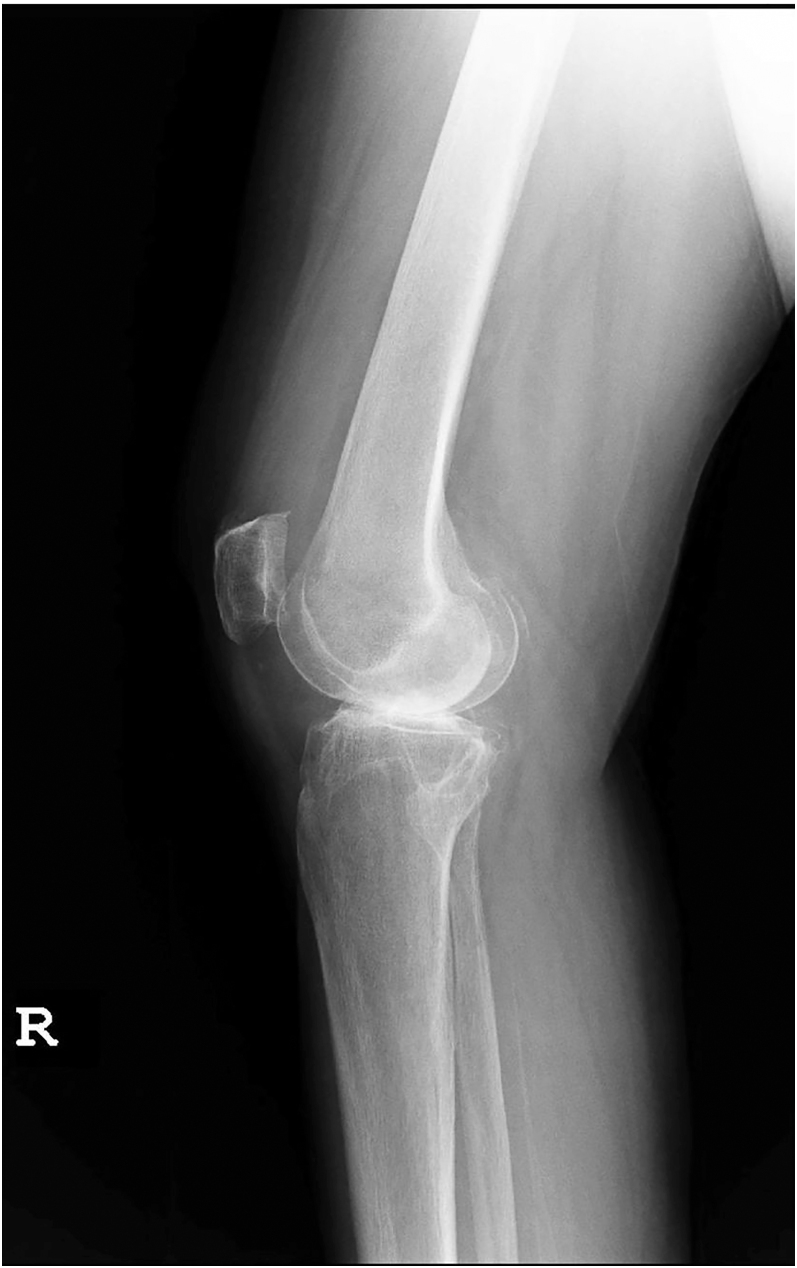
Sagittal plane imaging. Non-weight-bearing radiographs do not need to be full-length images.

The lateral view provides critical insights into the sagittal plane anatomy of the knee and does not require full-length images (Fig. 3). To obtain this view, the patient should lie on their side with the knee in slight flexion, allowing the radiographic beam to center on the joint. This non-weight-bearing view facilitates the evaluation of lateral femoral bowing, femoral condylar shaft angles, tibial bowing, tibial slope, and posterior condylar offset. Standardizing this view helps ensure consistency across varying levels of patient mobility and flexibility.

### Sunrise view (skyline)

**Figure 4:**
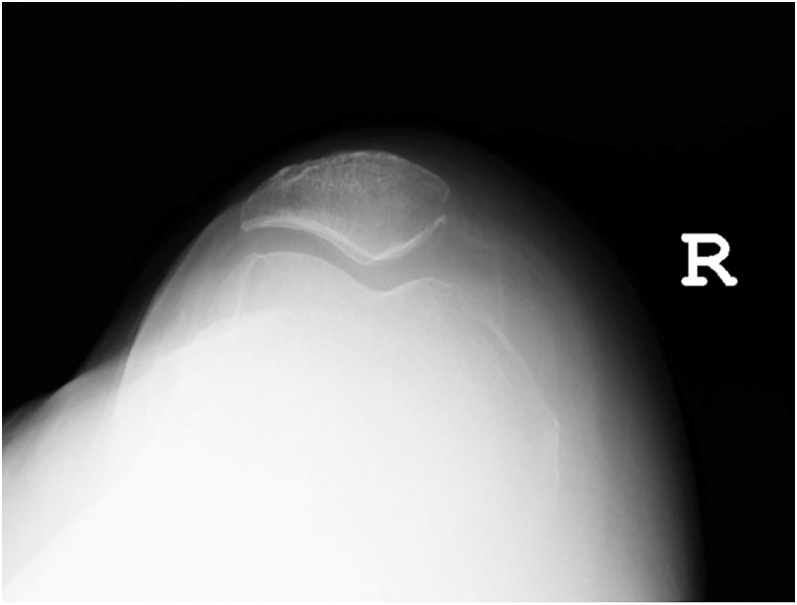
Sunrise or skyline view of the patellofemoral articulation.

The sunrise (or skyline) view is captured with the knee in flexion to evaluate the patellofemoral joint (Fig. 4). Although not used to measure alignment, this view remains crucial for assessing patellar tracking and identifying abnormalities, such as patellar dysplasia or maltracking. These findings can influence the surgical planning and the interpretation of radiographic alignment.

### Advanced imaging techniques

While three-dimensional imaging modalities, such as MRI and CT scans, provide enhanced anatomical detail, their use in limb alignment assessment is limited due to their non-weight-bearing nature. However, they are powerful tools for evaluating rotational alignment, bone morphology, and three-dimensional geometric changes when required. The use of these technologies demands strict standardization of acquisition protocols to ensure reproducibility and accuracy.

## Positioning considerations for radiographs

Proper positioning during radiographic image acquisition is essential to minimize measurement errors and ensure accurate assessment of limb alignment. Variations in limb positioning, particularly in full-length radiographs, can introduce angular errors that impact diagnostic interpretation and surgical planning.

### General setup & positioning

Angular measurement errors in full-length limb radiographs primarily stem from variations in limb positioning during image capture. To minimize these errors, patients should stand with their weight evenly distributed through both knees. The feet and ankles should be placed in a comfortable, natural position for balanced weight-bearing, typically 8–10 inches apart, with the knees fully extended.

It is important to note that closed-leg radiographs have also been utilized to investigate joint line obliquity (JLO) changes following TKA (24). While this method is not standard for assessing native limb alignment, surgeons should be aware of its use in specific clinical or research contexts.

### Rotation errors

Limb rotation during imaging is a common source of alignment error. Proper positioning of the knee’s flexion plane forward is essential to avoid rotational errors. However, reliance on specific anatomical landmarks, such as the patella or tibial tuberosity, can introduce inconsistencies, particularly in knees with significant structural variations.

### Recognizing setup rotations

Rotational setup errors can often be identified by examining frontal image profiles of the femur and tibia for symmetry loss. Even in patients with advanced disease, significant joint space loss, or severe malalignment, rotational variations can typically be recognized through key radiographic features.

**Figure 5:**
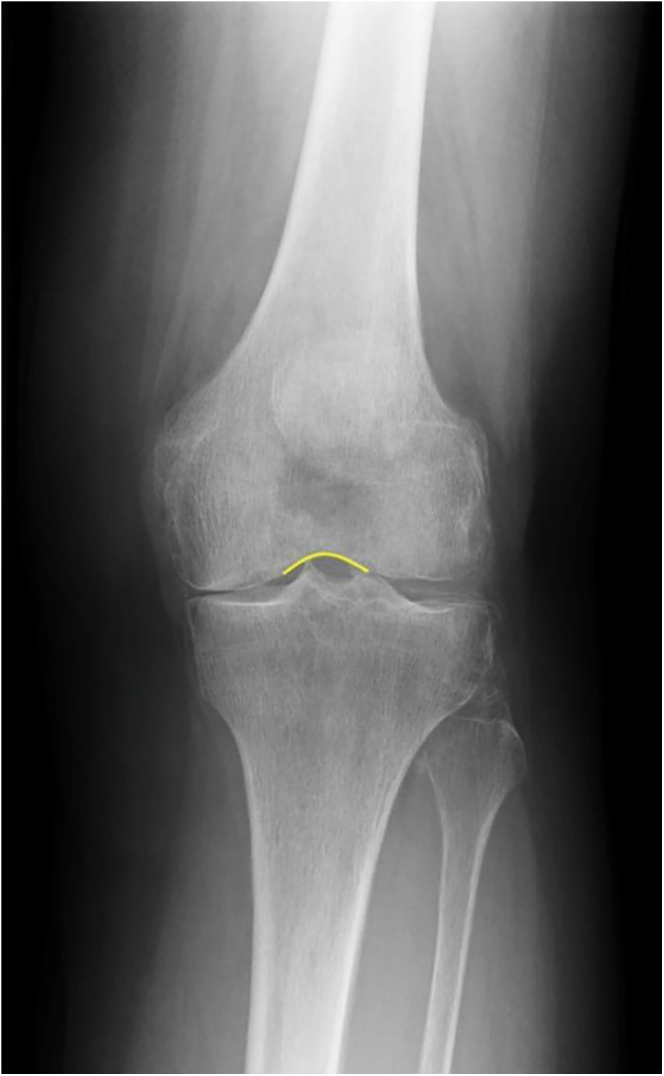
A well-positioned knee with symmetric notch slopes and condyles.

In a well-positioned knee, the frontal radiograph reveals distinct, symmetrical features:

- Symmetrical slopes of the intercondylar notch.
- Profile symmetry of the medial and lateral femoral condyles (as shown in Fig. 5).

Deviations from these features, such as changes in the condylar profiles or shifts in the patella outline, indicate internal or external rotation of the knee during image acquisition.

### Lateral view considerations

Lateral view radiographs provide critical information about the sagittal alignment of the knee. For a comprehensive evaluation, these images should include distal femoral and proximal tibial shafts.

To achieve an optimal lateral view, the patient is typically positioned side-lying with the knee in slight flexion. This alignment standardizes the bone planes with the coronal setup while ensuring image clarity. It is important to note that lateral views are not weight-bearing, which must be considered when interpreting sagittal alignment.

Key features assessed on lateral view radiographs include:

- Lateral femoral bowing.
- Femoral condylar shaft angle.
- Tibial bowing.
- Tibial slope.
- Posterior condylar offset.

### Key takeaways

- Proper positioning is crucial for obtaining reliable radiographs and minimizing alignment errors.
- Recognizing setup variations, particularly rotational errors, is critical for accurate interpretation and subsequent clinical and surgical planning.
- The use of standardized positioning techniques ensures consistency, enhances image quality, and facilitates more precise assessment of limb alignment.

## Current terms for axes and angles used to measure limb alignment

Standardized terminology for axes and angles is essential for accurately measuring and interpreting limb alignment. The following terms represent widely accepted definitions and measurements used in clinical practice and research.

### Hip-knee-ankle angle (HKA)

#### Mechanical HKA (mHKA)

The mechanical HKA (mHKA) angle is the primary radiological measurement of coronal alignment and reflects the relationship between the load-bearing axis of the limb and the orientation of the knee joint (25). It is defined as the angle between the femoral mechanical axis (FMA) (line from the center of the femoral head to the intercondylar notch center) and the tibial mechanical axis (TMA) (line from the tibial plateau center to the ankle joint center).

- An angle of 180° indicates a neutral alignment; the knee center is on the load-bearing axis (0° of deformity).
- An angle >180° indicates a valgus alignment; the knee center is medial to the load-bearing axis.
- An angle <180° indicates a varus alignment; the knee center is lateral to the load-bearing axis.

This value is referred to as the mechanical HKA (mHKA) to distinguish it from calculated alignment measurements. It refers to the actual load-bearing axis of the limb, incorporating the effects of arthritis, joint wear, deformity, or soft tissue imbalance, and can change significantly over time (25).

**Figure 6:**
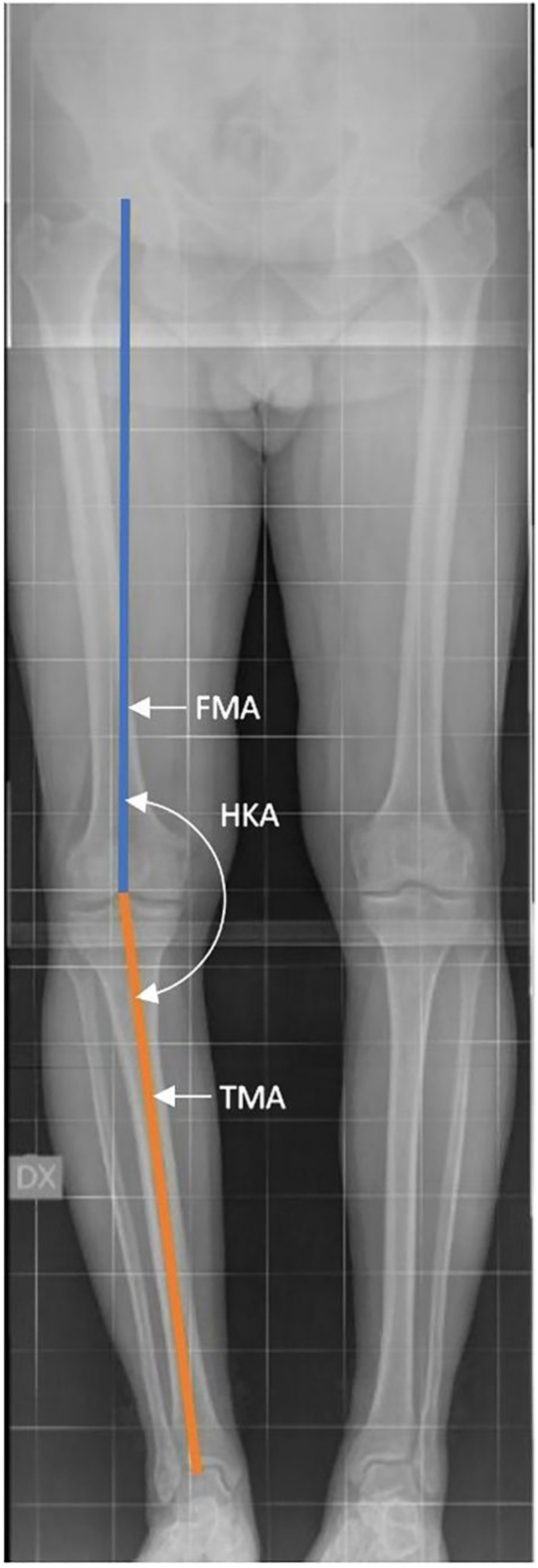
Mechanical HKA (mHKA) measures the angle between the FMA and the TMA.

Alternatively, the mHKA can also be measured in terms of angular deviation from 0°, using the angular relationship between the extension of the FMA and the TMA, as shown in Fig. 6:

- In a varus knee, the extension of the FMA falls laterally to the TMA, resulting in a negative mHKA (i.e., −3°, indicating 3° of varus).
- In a valgus knee, the extension of the FMA falls medially to the TMA, resulting in a positive mHKA (i.e., +2°, indicating 2° of valgus).
- If the TMA and the FMA are parallel, the alignment is considered neutral (0°).

PAS recommended using the first method (absolute angular value from hip to ankle) for measuring the mHKA, particularly for research purposes. In clinical practice, surgeons typically describe alignment in terms of degrees of varus or valgus deviation (e.g. 2° varus) (see Table 1).

**Table 1:** Mechanical HKA (mHKA) angle measurement and alternative methods.

mHKA angle	mHKA, alternative method	mHKA, alignment definition
…	-…	… varus
178°	−2°	2° varus
179°	−1°	1° varus
Neutral 180°	Neutral 0°	Neutral 0°
181°	+1°	1° valgus
182°	+2°	2° valgus
…	+…	… valgus

#### Arithmetic HKA (aHKA)

The arithmetic HKA (aHKA) is a calculated value that estimates the patient’s native or constitutional alignment of the lower limb, derived from bone geometry alone. It is determined by subtracting the mechanical lateral distal femoral angle (mLDFA) from the mechanical medial proximal tibial angle (mMPTA) (6, 25):

aHKA = mMPTA − mLDFA

Unlike mechanical HKA, the arithmetic HKA ignores the joint line convergence angle (JLCA), which can be altered by cartilage loss or joint subluxation in arthritic knees. In non-arthritic knees, the JLCA is usually minimal (mean −0.5°) and does not significantly affect aHKA (25).

- An aHKA of 0° indicates a neutral constitutional limb alignment.
- An aHKA <0° suggests a predominance of constitutional varus (larger mLDFA).
- An aHKA >0° indicates constitutional valgus (larger mMPTA).

Notably, recent studies have shown that multiple functional knee phenotypes may exist even within the same CPAK category (26), highlighting the importance of differentiating between mechanical and constitutional alignment during TKA planning.

### Mechanical lateral distal femoral angle (mLDFA)

**Figure 7:**
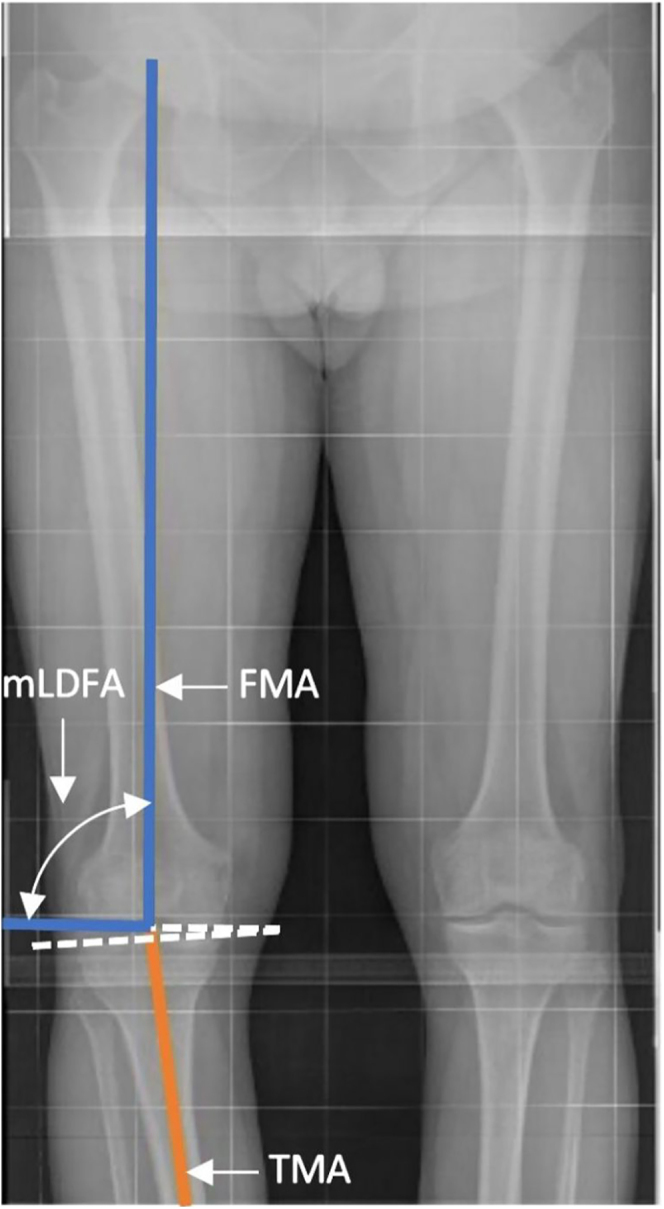
mLDFA is the lateral angle between the FMA and a line tangent to the femoral condyles.

The *mLDFA* is the lateral angle between the FMA and a line tangent to the distal femoral condyles (Fig. 7):

- An mLDFA of 90° indicates neutral alignment.
- An mLDFA>90° suggests a varus femur.
- An mLDFA <90° indicates a valgus femur.

It is important to distinguish the mechanical neutrality interpretation from population-based anatomical norms. Studies have shown that the mean mLDFA in non-arthritic knees is approximately 88°, which reflects normal anatomical variation, not mechanical neutrality (14). Therefore, while an mLDFA of 88° may be statistically normal, it would represent 2° of mechanical valgus, as it deviates from the 90° required to maintain a neutral load-bearing axis.

### Mechanical medial proximal tibial angle (mMPTA)

**Figure 8:**
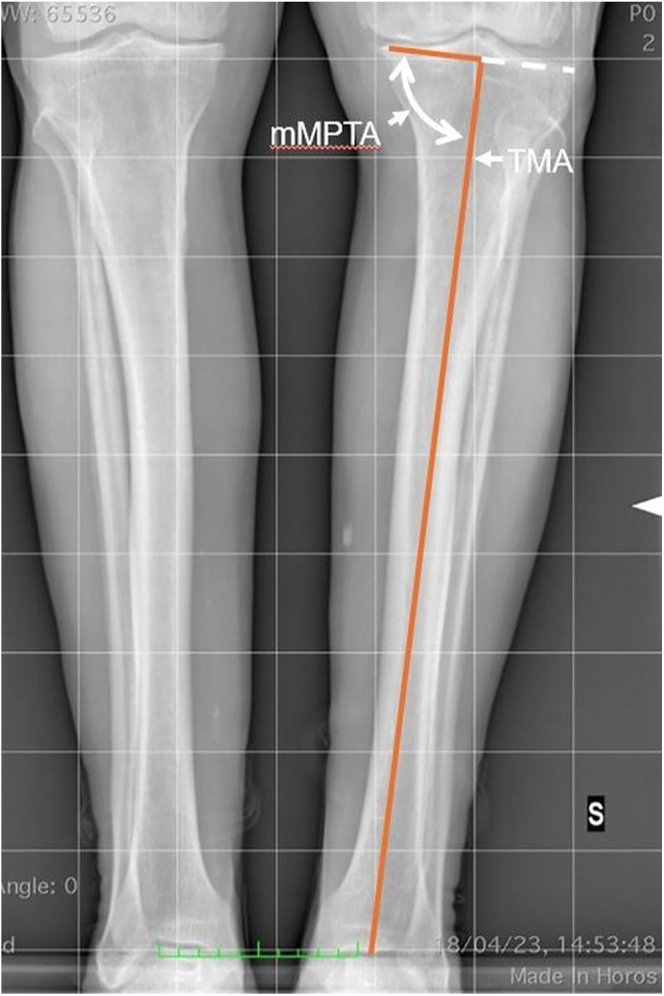
mMPTA is the medial angle between the TMA and the proximal tibia’s joint line.

The m*MPTA* is the medial angle between the TMA and the proximal tibial joint line (Fig. 8).

- An mMPTA of 90° indicates neutral alignment.
- An mMPTA >90° suggests a valgus tibia.
- An mMPTA <90° indicates a varus tibia.

Similarly, while the population mean mMPTA is approximately 87°, this reflects normal anatomy, not mechanical neutrality. In a mechanical alignment framework, an 87° tibia would be classified as 3° varus, which aligns with the concept of constitutional varus as described in the literature (6, 9, 14).

### Joint line convergence angle (JLCA)

**Figure 9:**
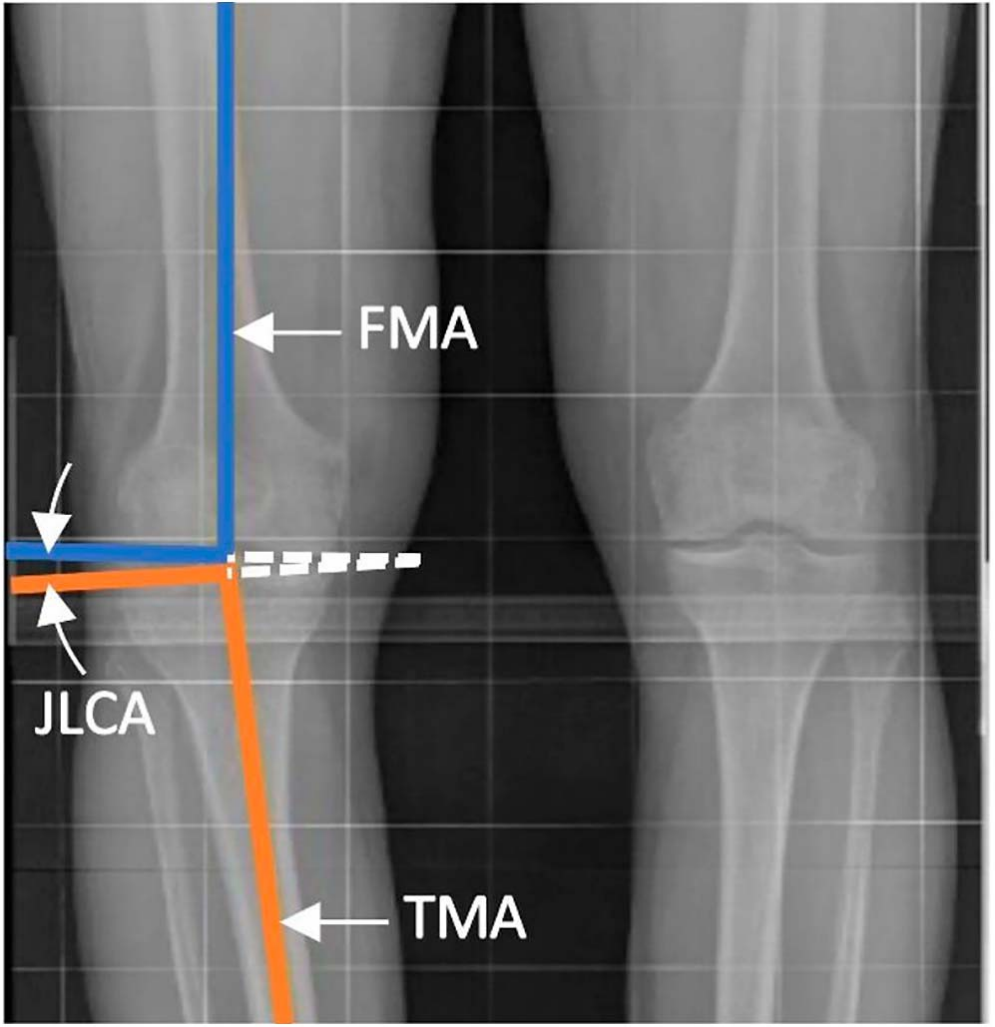
JLCA is formed between the joint line of the distal femur and proximal tibia.

The JLCA is the angle formed between the distal femur and proximal tibial joint line (Fig. 9):

- Positive values occur medially (varus convergence).
- Negative values occur laterally (valgus convergence).

The JLCA accounts for joint space narrowing due to cartilage wear, bone loss, or collateral ligament insufficiency in arthritic knees. Knee alignment incorporating JLCA can be calculated as:

Knee alignment = 180° − (FMA + TMA ± JLCA)

Examples:

- Varus knee: knee alignment = 180° − (93° + 88° − 5°) = 176° = 4° varus.
- Valgus knee: knee alignment = 180° − (95° + 90° + 5°) = 170° = 10° valgus.

### Femoral anatomical-mechanical angle (AMA) or valgus correction angle (VCA)

**Figure 10:**
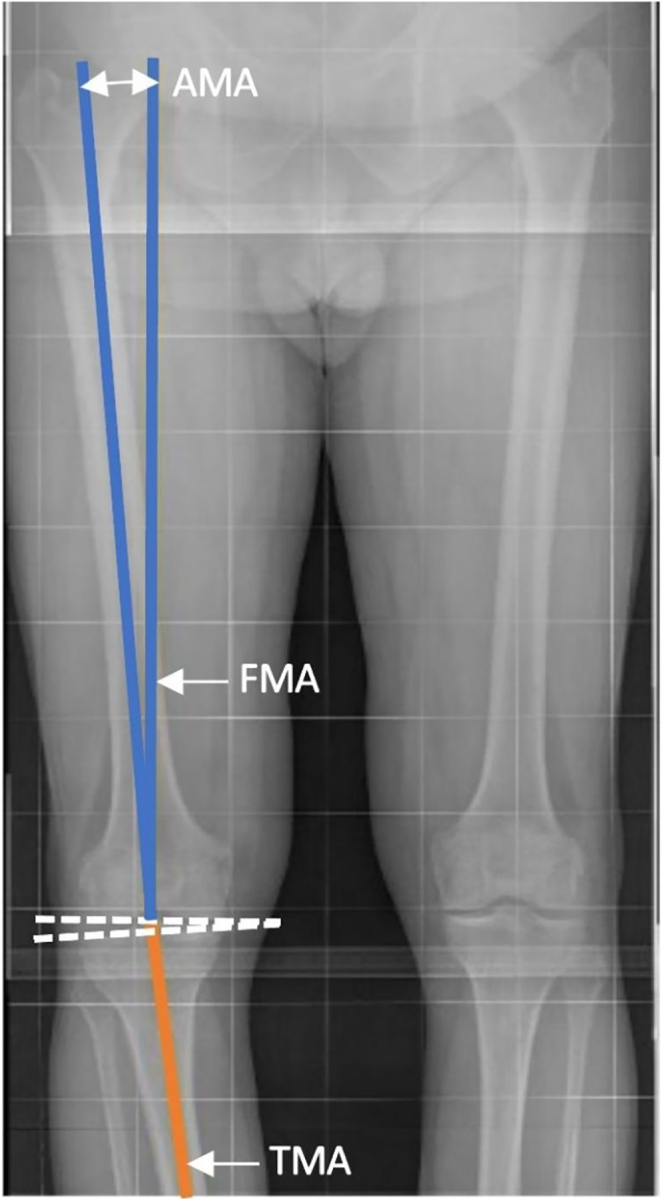
AMA is the angle between the distal anatomical femoral shaft line and the FMA.

The AMA (or VCA) is the angle between the distal anatomical femoral shaft line and the FMA. It is particularly relevant for neutralizing the mLDFA in systematic alignments (Fig. 10).

### Tibial anatomical mechanical angle (TAMA)

**Figure 11:**
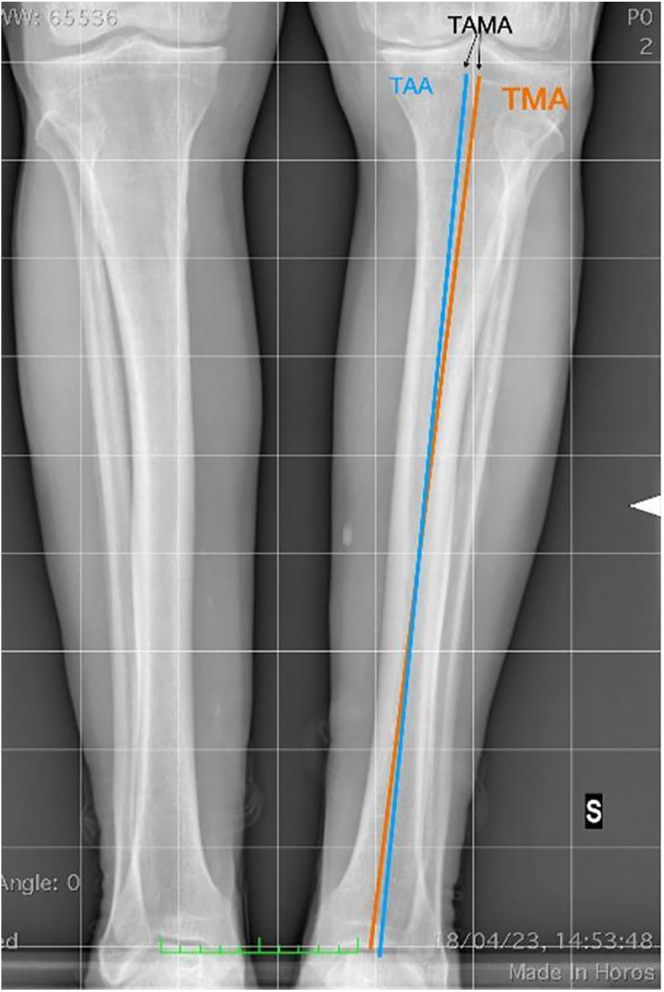
TAMA is the angle between the tibial mechanical and anatomical axes (usually coincident axes).

The TAMA is the angle between the TMA and the tibial anatomical axis (Fig. 11). These axes are usually coincidental. The tibial anatomical axis is typically defined by connecting the midpoints of the diaphysis at the junctions of the upper and middle thirds and the middle and lower thirds of the tibia on a full-length radiograph. A discrepancy between the two axes indicates the presence of an extra-articular deformity (Fig. 11).

### Joint line obliquity (JLO)

JLO describes the inclination of the joint line relative to the lower limb axis. Although often described as ‘varus’ or ‘valgus’, JLO is independent of overall limb alignment.

According to the CPAK classification, the JLO is defined as:

- Neutral: joint line perpendicular to the load-bearing axis.
- Apex proximal: joint line inclined toward the proximal aspect.
- Apex distal: joint line inclined toward the distal aspect.

JLO is calculated:

JLO = mMPTA + mLDFA

- A value of 180° indicates a neutral joint line.
- A value >183° indicates an apex proximal joint line.
- A value <177° indicates an apex distal joint line.

### Required landmarks for measurement

Accurate limb alignment measurements rely on the following anatomical landmarks:

- Center of the hip
- Center of the intercondylar notch
- Center of the tibial plateau
- Center of the ankle
- Femoral joint line
- Tibial joint line
- Ankle joint line

### Notes

Arthroplasty primarily addresses intra-articular changes, focusing on restoring joint alignment and function. However, alteration to the joint line caused by bone loss can significantly affect all angles associated with the affected bone. This classification does not encompass extra-articular deformities. For example, in true tibia vara, where the deformity apex is located at the tibial junctions, only the resulting TAMA difference is considered within this system. The unique characteristics and angles of extra-articular deformities are not included.

## Conclusion

Restoring limb alignment is fundamental to reintegrating osteoarthritic patients into an active, pain-free lifestyle. Modern surgical techniques, evolving beyond traditional mechanical alignment, play a crucial role in achieving this goal. Mastery of these approaches – along with a deep understanding of the terminology, principles, and execution of limb alignment strategies – is essential for advancing orthopedic practice and improving patient outcomes.

The proposed PAS nomenclature provides a much-needed framework for standardizing discussions and practices within the orthopedic community. This initiative aims to disseminate knowledge, clarify complex concepts, and foster meaningful engagement, research, and innovation.

Central to this effort is refining the definition of limb alignment and its key descriptors. The descriptors included in this initiative are intentionally focused on essential elements, offering a practical framework for understanding and applying alignment techniques. While other descriptive lines exist, their omission reflects the need for clarity and practicality rather than their lack of importance. Variations in the described techniques often represent refinements rather than novel approaches, which aligns with the project’s overarching goal of building upon established foundations.

As orthopedic evaluation evolves from traditional two-dimensional (2D) analyses to advanced three-dimensional (3D) assessments, the adaptability of fundamental knee angles remains critical. Baseline measurements such as the mMPTA and mLDFA allow for calculating the aHKA and the subsequent use of the Coronal Plane Alignment of the Knee (CPAK) classification. These measurements, while not inherently required for alignment strategies, provide valuable insights for preoperative evaluation and the selection of optimal surgical techniques.

Looking ahead, the integration of advanced technologies such as large language models and deep learning algorithms offers tremendous potential for transforming orthopedic practice. Although challenges remain in assessing their precise value, these tools are expected to play an increasingly integral role in orthopedic research, diagnostics, and clinical workflows. Embracing standardization efforts, such as the PAS framework, is essential for harnessing these advancements to enhance patient care and promote innovation in orthopedic science.

## ICMJE Statement of Interest

The authors declare that there is no conflict of interest that could be perceived as prejudicing the impartiality of the research reported.

## Funding Statement

This research received no specific grant from funding agencies in the public, commercial, or not-for-profit sectors.
